# Evaluation of deep learning‐based auto‐segmentation algorithms for delineating clinical target volume and organs at risk involving data for 125 cervical cancer patients

**DOI:** 10.1002/acm2.13097

**Published:** 2020-11-25

**Authors:** Zhi Wang, Yankui Chang, Zhao Peng, Yin Lv, Weijiong Shi, Fan Wang, Xi Pei, X. George Xu

**Affiliations:** ^1^ Center of Radiological Medical Physics University of Science and Technology of China Hefei China; ^2^ Department of Radiation Oncology First Affiliated Hospital of Anhui Medical University Hefei China; ^3^ Anhui Wisdom Technology Co., Ltd. Hefei Anhui China

**Keywords:** auto‐segmentation, clinical target volumes, deep learning, organs at risk

## Abstract

**Objective:**

To evaluate the accuracy of a deep learning‐based auto‐segmentation mode to that of manual contouring by one medical resident, where both entities tried to mimic the delineation "habits" of the same clinical senior physician.

**Methods:**

This study included 125 cervical cancer patients whose clinical target volumes (CTVs) and organs at risk (OARs) were delineated by the same senior physician. Of these 125 cases, 100 were used for model training and the remaining 25 for model testing. In addition, the medical resident instructed by the senior physician for approximately 8 months delineated the CTVs and OARs for the testing cases. The dice similarity coefficient (DSC) and the Hausdorff Distance (HD) were used to evaluate the delineation accuracy for CTV, bladder, rectum, small intestine, femoral‐head‐left, and femoral‐head‐right.

**Results:**

The DSC values of the auto‐segmentation model and manual contouring by the resident were, respectively, 0.86 and 0.83 for the CTV (*P* < 0.05), 0.91 and 0.91 for the bladder (*P* > 0.05), 0.88 and 0.84 for the femoral‐head‐right (*P* < 0.05), 0.88 and 0.84 for the femoral‐head‐left (*P* < 0.05), 0.86 and 0.81 for the small intestine (*P* < 0.05), and 0.81 and 0.84 for the rectum (*P* > 0.05). The HD (mm) values were, respectively, 14.84 and 18.37 for the CTV (*P* < 0.05), 7.82 and 7.63 for the bladder (*P* > 0.05), 6.18 and 6.75 for the femoral‐head‐right (*P* > 0.05), 6.17 and 6.31 for the femoral‐head‐left (*P* > 0.05), 22.21 and 26.70 for the small intestine (*P* > 0.05), and 7.04 and 6.13 for the rectum (*P* > 0.05). The auto‐segmentation model took approximately 2 min to delineate the CTV and OARs while the resident took approximately 90 min to complete the same task.

**Conclusion:**

The auto‐segmentation model was as accurate as the medical resident but with much better efficiency in this study. Furthermore, the auto‐segmentation approach offers additional perceivable advantages of being consistent and ever improving when compared with manual approaches.

## 1 Introduction

Cervical cancer is one of the most common malignant tumors in the female reproductive system. The incidence and mortality rates of cervical cancer rank the fourth highest among all female cancer patients.^1^ Radiation treatment (RT) is an effective method for cervical cancer treatment,^2^ and the mainstream technology today is based on the concept of intensity‐modulated radiation therapy (IMRT). In radiotherapy planning, the precise delineation of the clinical target volume (CTV) and organs at risk (OARs) is essential in ultimately delivering the necessary amount of radiation dose to the target area while sparing adjacent normal tissues from complications. Manual delineation of the OARs, however, is time‐consuming and labor‐intensive in the RT planning workflows. Studies have shown that as much as 120 min can be required for a clinician to manually delineate the OARs of a cervical cancer patient.^3^ Inter‐observer variability (IOV) has been found among radiation oncologists who perform manual contours, and even the same physician can have different manual contours at different times due to fatigue and other factors.^4^, ^5^, ^6^, ^7^, ^8^ The variability can lead to a higher error level than the planning and setup errors.^9^, ^10^, ^11^, ^12^

Automatic segmentation of CTV and OARs can alleviate physicians' burden and reduce variability. To that end, atlas‐based approaches have been reported.^13^, ^14^, ^15^ However, the atlas‐based auto‐segmentation methods require users to establish their own templates, and the subsequent applications can suffer from the large number of patient cases in the template and the poor accuracy of manual contouring. Moreover the image processing of the atlas‐based auto‐segmentation requires a long time. These issues limit further development of this technology.

In recent years, convolutional neural networks (CNNs) have been proven to be an effective tool in auto‐segmentation of the CTV and OARs of the head and neck,^16^, ^17^, ^18^, ^19^ thoracic cavity,^20^, ^21^, ^22^, ^23^ abdomen,^24^, ^25^, ^26^ and pelvis.^27^, ^28^, ^29^, ^30^ Studies have shown that for auto‐segmentation of OARs in head and neck cancers and chest cancers, the accuracy of deep learning‐based auto‐segmentation^19^, ^21^, ^26^, ^31^ is significantly higher than that of the atlas‐based method.^32^, ^33^, ^34^ Men et al.^30^ used deep‐dilated CNNs to yield more accurate segmentation results in delineating the CTV and OARs of rectal cancer patients. Liu et al.^35^ used the modified U‐Net model for auto‐segmentation of OARs of cervical cancer, and the evaluation of radiation oncologists showed that the results predicted by the model were highly consistent with those of the radiation oncologists. Wong et al^.^ ^36^ verified that the accuracy of deep learning‐based auto‐segmentation is comparable to that of expert inter‐observer variability for RT structures and suggested that the use of deep learning‐based models in clinical practice would likely realize significant benefits in RT planning workflow and resources.

However, most previous studies^19^, ^24^, ^25^, ^30^ have focused on the accuracy of auto‐segmentation ignoring the evaluation of learning ability in the clinical practice. This study aims to compare the learning abilities of the auto‐segmentation model and a medical resident — both learned from the same senior radiation oncologist. Higher accuracy represents higher learning ability, and smaller variance corresponds to better stability. We first collected cervical cancer cases delineated by the same senior radiation oncologist. Next, the testing cases were delineated separated by a medical resident under the instruction by the senior physician for 8 months and by the auto‐segmentation model trained on the training set. The auto‐segmentation model was compared against the medical resident using the remaining 25 cases in the testing set.

## 2 MATERIALS AND METHODS

### 2.A Datasets

We retrospectively collected 125 cases of cervical cancer receiving IMRT between January 2019 and May 2020 at the First Affiliated Hospital of Anhui Medical University in China. These female patients were between 22 and 86 yr of age, with an average age of 53.8 yr. The CT scanning covered from the lower lumbar spine to the sciatica knot and pelvic cavity. The CT slice thickness was 5 mm. The CT image datasets were transmitted to the Eclipse 13.6 treatment planning system (TPS).

The manual delineation of the cervical cancer CTV was conducted in accordance with the guidelines of by the Radiation Therapy Oncology Group (RTOG).^37^ The senior radiation oncologist manually contoured the CTV and OARs on the Eclipse TPS according to the International Commission on Radiation Units and Measurements (ICRU) report 50.^38^ The CTV starts from the bifurcation of the common iliac artery and includes the primary tumor, uterus, appendix, part of the vagina (the upper half or two‐thirds of the vagina according to the primary tumor), and pelvic lymph nodes (common iliac, external iliac, internal iliac, obturator, and presacral).

### 2.B Deep learning‐based auto‐segmentation

**Fig 1:**
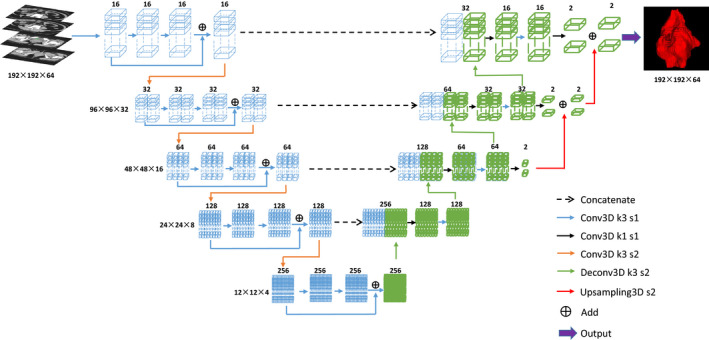
Structure of the network model.

In this study, we investigated the use of a 3D CNN for delineating CTVs and OARs of cervical cancers. As shown in Fig. 1, the network consists of an encoder which extracts features from data and a decoder which performs the pixel‐wise classification. The encoder consists of five successive residual blocks. Each block contains three convolution layers with 3 × 3 × 3 kernel, and there is a spatial dropout layer between the early two convolution layers to prevent the network from overfitting. Spatial down‐sampling is performed by a convolution layer with 3 × 3 × 3 kernel and 2 × 2 × 2 stride. The decoder consists of four successive segmentation blocks. Each block contains two convolution layers with the kernel of 1 × 1 × 1 and 3 × 3 × 3, respectively. Spatial up‐sampling is performed by a deconvolution layer with 3 × 3 × 3 kernel and 2 × 2 × 2 stride. Here each convolution layer is followed by an instance normalization, and a leaky rectified linear unit. Four dashed arrows in the Fig. 1 indicate four skipping connections that copy early feature‐maps and concatenate them with later feature‐maps that have the same size to preserve high‐resolution features. In the final three segmentation blocks, a 1 × 1 × 1 convolution layer is used to map the feature tensor to the probability tensor with the two channels, before all results are merged by the up‐sampling operation to enhance the precision of segmentation results. Finally, a SoftMax activation is used to output a probability of each class for every voxel. The network has achieved high precision in segmentation of thoracic and abdominal organs, which has been validated in previous research by Peng et al.^39^ and integrated into DeepViewer (commercial auto‐segmentation software based on deep learning). ^40^, ^41^

This study included 125 cervical cancer cases, 100 of which were randomly selected and divided into training and validation sets at a ratio of 4:1, while the remaining 25 cases were used to test the model. The weighted DSC was selected as the loss function, and Adam was selected as the optimizer. During training, data augmentation and deep supervision were used to avoid overfitting. The entire training process used the Python deep learning library Keras^42^ with TensorFlow^43^ as the backend, and a Nvidia Geforce RTX 2080Ti GPU card with 11G memory was used to train the model.

### 2.C Experiment

**Fig 2:**
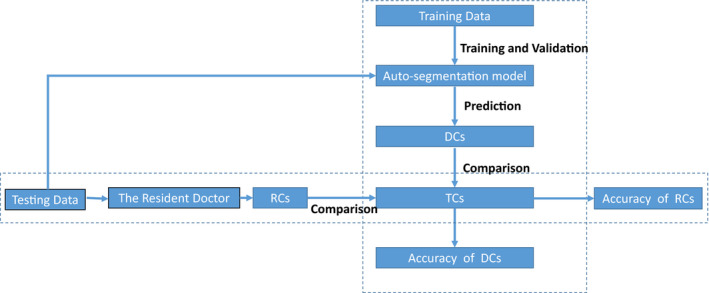
Flowchart of the experiment. DCs: Deep learning‐based auto‐segmentation contours; TCs: True contours delineated by the senior physician. RCs: Manual contours delineated by the medical resident.

To study the difference in learning ability between the auto‐segmentation model and the medical resident in delineating the CTVs and OARs, the auto‐segmentation model and the resident both learned from the same senior physician. The learning abilities of the auto‐segmentation model and the resident were evaluated by comparing the accuracy of the auto‐segmentation model and the resident in the 25 testing cases. The delineation objects included the CTV, bladder, femoral‐head‐right, femoral‐head‐left, small intestine, and rectum in cervical cancer. A flowchart of the experiment is shown in Fig. 2.

First, this study included 125 cervical cancer cases whose CTV and OAR contours were manually delineated by the same senior physician with 20 yr of clinical experience according to the above principles, and these contours were regarded as true contours (TCs) in this study.

Second, a medical resident, who was a student of the senior physician and had spent 8 months of training on how to delineate the CTV and OARs, was invited to participate in this experiment. Based on his experience, the medical resident independently delineated the CTVs and OARs of the 25 cervical cancer cases in the testing set layer by layer on the Eclipse TPS. During the delineation process, no time limit was applied, and the medical resident could not view anatomy books and online guidance, consult other doctors, or refer to previous cases. Under these circumstances, we obtained the resident contours (RCs) manually delineated by the medical resident.

Then, the auto‐segmentation model was trained on the 100 training cases delineated by the same senior physician. For training, the learning rate starts from 0.0005 and is divided by 10 when the validation loss does not significantly decrease in 10 successive epochs. The training process stops automatically when the validation loss does not decrease in 30 successive epochs. The trained model was integrated into DeepViewer. The deep learning‐based auto‐segmentation contours (DCs) of the CTVs and OARs for the 25 cases were obtained using DeepViewer.

Finally, to compare the learning abilities of the auto‐segmentation model and the medical resident, the accuracy of the deep learning‐based auto‐segmentation model and the accuracy of the medical resident were calculated, and paired Student's *t*‐tests were used for statistical analysis.

### 2.D Evaluation metrics

The DSC and HD were used to evaluate the accuracy of the auto‐segmentation model and the accuracy of the resident. The DSC is defined as follows:

(1) $$DSC=\frac{2\left|A\cap B\right|}{\left|A\right|+\left|B\right|},$$

where A is the DCs or RCs, and B is the TCs in our study. The numerator is twice as large as the intersection of A and B, and the denominator is the sum of A and B. A larger DSC corresponds to a higher degree of coincidence between the DCs or RCs and the TCs. The DSC ranges from 0 to 1, with the latter value indicating perfect performance.

The HD is defined as follows:

(2) $$HD\left(A,B\right)=\max\left(h\left(A,B\right),h\left(B,A\right)\right)$$

(3) $$h\left(A,B\right)=\max_{b\in B}\left(\min_{a\in A}∥a‐b∥\right)$$

where h(A,B) is the greatest of all the distances from a point in A to the closest point in B. A smaller value usually represents better segmentation accuracy.

## 3 RESULTS

**Fig 3:**
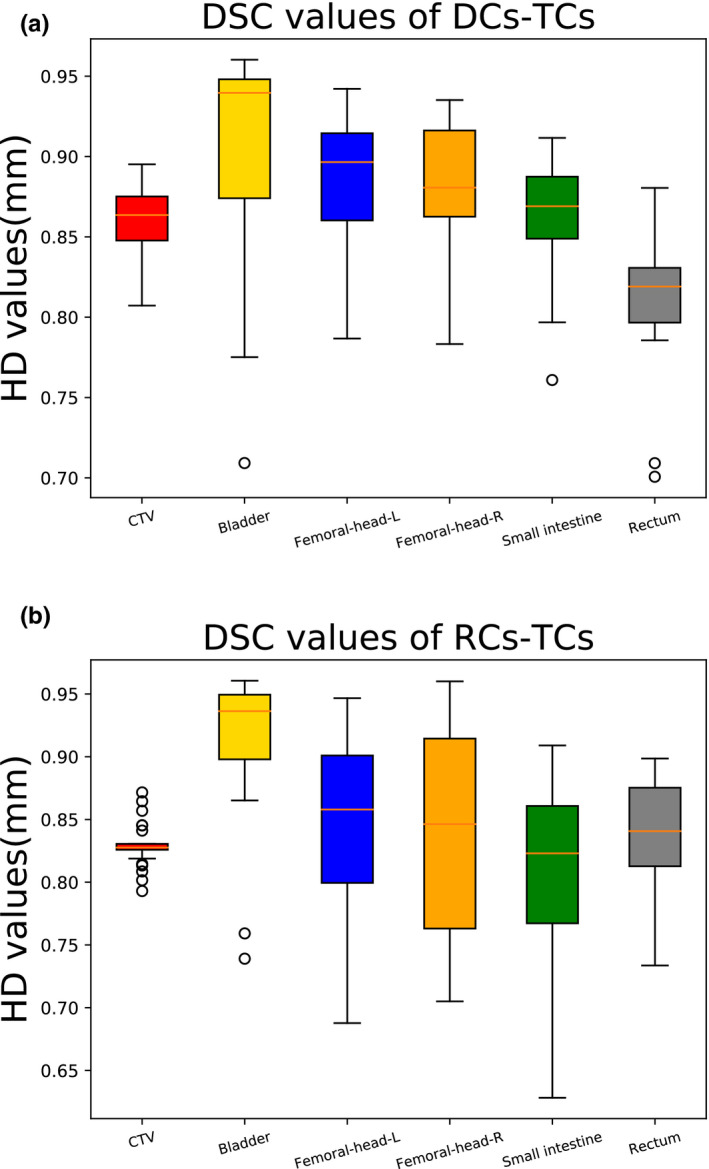
Boxplots obtained for DSC analyses. (a) DSC values of DCs‐TCs; (b) DSC values of RCs‐TCs.

The DSC values of deep learning‐based auto‐segmentation (the DSC of DCs‐TCs) and the DSC values of manual contouring by the resident (the DSC of RCs‐TCs) are summarized in Table 1 and displayed in Fig. 3. As shown in Table 1, the DSC values of the auto‐segmentation model for the CTVs and OARs were (CTV: 0.86 ± 0.02; bladder: 0.91 ± 0.06; femoral‐head‐left: 0.88 ± 0.05; femoral‐head‐right: 0.88 ± 0.04; small intestine: 0.86 ± 0.04; and rectum: 0.81 ± 0.06). Compared with the resident, the auto‐segmentation model had better accuracy for the CTV, femoral‐head‐left, femoral‐head‐right, and small intestine, and a significant difference was identified between auto‐segmentation model and the resident (*P* < 0.05). The auto‐segmentation model and the resident had comparable accuracy for the bladder, with DSC values of 0.91 ± 0.06 for both. For the rectum, the DSC value of the auto‐segmentation model was 0.03 lower than that of the resident, but no significant difference was observed (*P* > 0.05).

**Table 1:** DSC values of DCs‐TCs and RCs‐TCs.

	DCs‐TCs	RCs‐TCs	*P* value
CTV	0.86 ± 0.02	0.83 ± 0.02	<0.001
Bladder	0.91 ± 0.06	0.91 ± 0.06	0.684
Femoral‐head‐right	0.88 ± 0.05	0.84 ± 0.07	0.032
Femoral‐head‐left	0.88 ± 0.04	0.84 ± 0.08	0.025
Small intestine	0.86 ± 0.04	0.81 ± 0.07	0.002
Rectum	0.81 ± 0.04	0.84 ± 0.05	0.059

**Fig 4:**
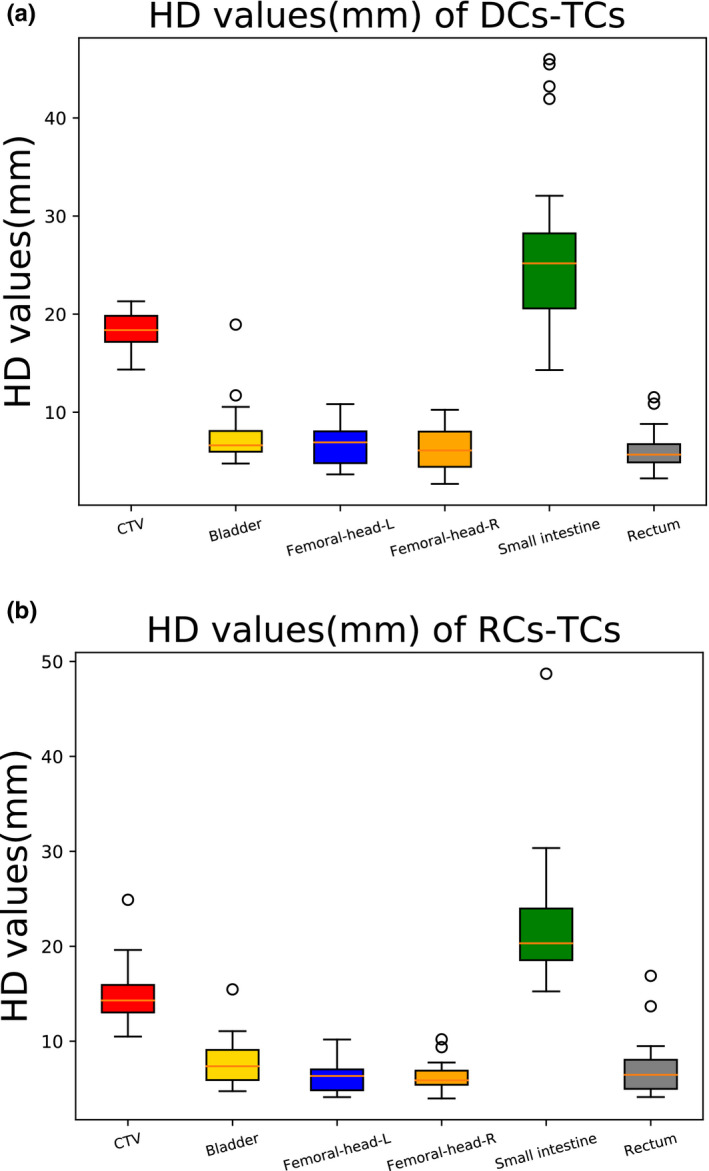
Boxplots obtained for HD analyses. (a) HD values of DCs‐TCs; (b) HD values of RCs‐TCs.

The HD values of deep learning‐based auto‐segmentation (the HD of DCs‐TCs) and the HD values of manual contouring by the resident (the HD of MCs‐TCs) are shown in Table 2 and Fig. 4. As shown in Table 2, the HD values of the auto‐segmentation model for the CTVs and OARs were (CTV: 14.84 mm ± 2.92 mm; bladder: 7.82 mm ± 2.42 mm; femoral‐head‐left: 6.18 mm ± 1.51 mm; femoral‐head‐right: 6.17 mm ± 1.15 mm; small intestine: 22.21 mm ± 6.64 mm; and rectum: 7.04 mm ± 2.88 mm). Compared with the resident, the auto‐segmentation model had better similarity for the CTV, and a significant difference was noted between the auto‐segmentation model and the resident (*P* < 0.05). The auto‐segmentation model had an accuracy comparable to the resident for the bladder, rectum, femoral‐head‐left, femoral‐head‐right, and small intestine.

**Table 2:** HD values (mm) for DCs‐TCs and RCs‐TCs.

	DCs‐TCs	RCs‐TCs	*P* value
CTV	14.84 ± 2.92	18.37 ± 1.59	<0.001
Bladder	7.82 ± 2.42	7.63 ± 2.88	0.813
Femoral‐head‐right	6.18 ± 1.51	6.75 ± 2.05	0.281
Femoral‐head‐left	6.17 ± 1.15	6.31 ± 2.12	0.789
Small intestine	22.21 ± 6.64	26.70 ± 8.76	0.051
Rectum	7.04 ± 2.88	6.13 ± 1.93	0.208

**Fig 5:**
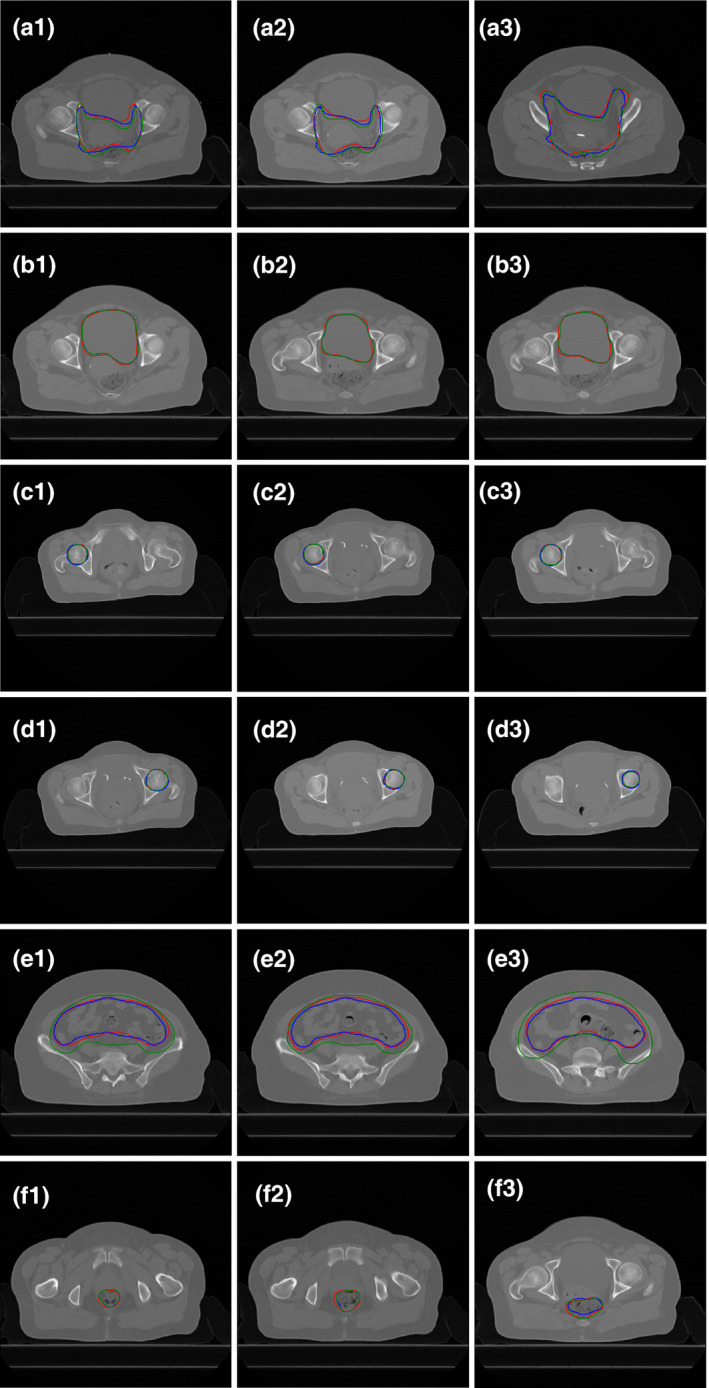
Results for best case in auto‐segmentation of CTV shown in CT slices. Red lines: Manual contours delineated by the senior physician (TCs); Green lines: Manual contours delineated by the medical resident (RCs); Blue lines: Deep learning‐based contours (DCs). (a) CTV; (b) Bladder; (c) Femoral‐head‐L; (d) Femoral‐head‐R; (e) Small intestine; (f) Rectum. The DSC values of DCs‐TCs and RCs‐TCs are 0.90 vs 0.86, 0.96 vs 0.96, 0.94 vs 0.93, 0.94 vs 0.95, 0.87 vs 0.80, and 0.82 vs 0.88, respectively.

The combined results of the DSC and HD show that the deep learning‐based auto‐segmentation model had better accuracy than the medical resident in delineating the CTV, with significant differences in both the DSC and HD (*P* < 0.05). As shown in Fig. 5 (panels a1–a3), deep learning‐based auto‐segmentation was more similar to the contours delineated by the senior physician. As shown in Fig. 5 (panels b1–b3, d1–d3 and e1–e3), the auto‐segmentation model and the medical resident performed comparably for the bladder, femoral‐head‐left, and femoral‐head‐right, with no significant differences. Regarding delineation of the small intestine and rectum (Fig. 5), the auto‐segmentation model was slightly better than the resident for the small intestine, and the medical resident was slightly better than the auto‐segmentation model for the rectum.

In terms of time requirements, the average time for delineation of a case, including the data pre‐processing and post‐processing steps, with the deep learning‐based auto‐segmentation model was approximately 2 min. Approximately 90 min was required for the medical resident to delineate a case manually, showing that the efficiency of auto‐segmentation is substantially higher than that of the medical resident.

## 4 DISCUSSION

In recent years, an increasing number of deep learning‐based methods have been applied to the field of medical imaging, which have great application potential in disease diagnosis,^44^, ^45^, ^46^, ^47^ lesion recognition,^48^, ^49^, ^50^, ^51^, ^52^ and image segmentation.^28^, ^52^, ^53^, ^54^ Especially in image segmentation, accuracy has always been the focus of attention. Most previous studies developed several superior models to improve the accuracy of auto‐segmentation, and some studies compared the accuracy of different auto‐segmentation models.^19^, ^24^, ^25^, ^30^ In this study, we investigated the use of the deep learning‐based auto‐segmentation model to delineate the CTVs and OARs of cervical cancer cases and conducted a comparative analysis with the manual delineation results of a medical resident. The auto‐segmentation model and the medical resident learned from the same senior clinical doctor to avoid the impact of different physicians. The auto‐segmentation model learned the contours of the CTVs and OARs manually delineated by the senior physician for training, and the medical resident mimicked the senior physician how to delineate the CTV and OARs. In this situation, the comparison between the auto‐segmentation model and the medical resident is meaningful. The learning abilities of the auto‐segmentation model and the medical resident could be evaluated by comparing the accuracy of the auto‐segmentation model and the accuracy of the medical resident.

The accuracy of the deep learning‐based auto‐segmentation model was found to be higher than that of the medical resident in delineating the CTVs and most OARs of cervical cancer. The accuracy of auto‐segmentation in this study is comparable to those in similar studies and even higher for some OARs,^19^, ^24^, ^28^, ^30^, ^35^, ^36^ and the reason may be that the cases in this study were delineated by the same senior physician. The auto‐segmentation model is more accurate in the delineation of the CTV, small intestine, femoral‐head‐right, and femoral‐head‐left. The boundaries of the CTV and small intestine in cervical cancer are not clear, and the resolution of soft tissue in CT images is not good. The senior physician must delineate the contours according to the actual situation of the patient; at this point, the resident lacks sufficient knowledge and experience. Moreover the location of the small intestine in CT images is different from the location of the small intestine during radiation treatment. To better protect the small intestine with as low a dose as possible, the senior physician often uses a larger outline containing the small intestine as the contours of the small intestine. In other words, the actual outline of the small intestine will be slightly expanded relative to the original contours. In this case, the auto‐segmentation model is more likely to learn the contouring standards and experience of the senior physician in delineating the CTV and the small intestine, while a medical resident with only 8 months of internship experience does not possess this ability. For the femoral‐head‐right and femoral‐head‐left, the boundaries are clear, and the auto‐segmentation model can easily recognize and delineate the contour boundary with high precision.

For the bladder and rectum, the performance of the auto‐segmentation was comparable to that of the medical resident. The boundary of the bladder is clearer, with no external expansion. The medical resident can easily delineate the contours of the bladder through CT images and his own knowledge, resulting in no significant difference between auto‐segmentation and the medical resident. For the rectum, the accuracy of the auto‐segmentation model in this study was comparable to that of auto‐segmentation in previous studies,^19^, ^28^, ^35^, ^36^ but the accuracy of the medical resident was more similar to that of the senior physician, which may be due to the small size of the rectum and the low resolution of the rectum on CT images. The auto‐segmentation model had poor predictive ability for small volumes and did not recognize some of the layers; thus, related research will be carried out to address this problem in the future.

In this study, the deep learning‐based auto‐segmentation model was found to be as accurate as the resident, and the auto‐segmentation model had better stability, indicating that the deep learning‐based auto‐segmentation model reached or even exceeded the level of the resident. In terms of time requirements, the auto‐segmentation model was better than the resident (2 and 90 min for a patient’s CTV and OARs, respectively). In many clinical situations, the CTV and OARs are first delineated by a resident, and a senior physician modifies the contours based on the resident’s results. According to the results of our experiment, the auto‐segmentation model can even replace part of the work of residents. Senior physicians modify the contours based on auto‐segmentation directly and obtain contours acceptable for clinical radiotherapy, which can improve clinical efficiency. On the other hand, residents are not required to delineate all cases during their internship. They can delineate select cases to gain relevant experience and have more time to learn and think, which can reduce the burden of residents. Finally, the auto‐segmentation model may change the traditional clinical delineation pattern. Residents modify the contours based on auto‐segmentation, and senior physicians modify the contours based on the residents’ results for clinical therapy. In this process, the auto‐segmentation model can help residents learn more information about the contouring habits and standards of senior physicians. In short, the deep learning‐based auto‐segmentation model has considerable potential for development, and the use of these models in clinical practice will improve the efficiency of clinical residents and the accuracy of the contouring of residents.

Some limitations exist in this study. First, whether the use of the auto‐segmentation model will reduce the delineation accuracy of senior physicians is uncertain. Second, whether the model trained on a local hospital data can be effectively applied to other hospitals requires further investigation.

## 5 CONCLUSION

In this study, we compared and analyzed differences in learning ability between the deep learning‐based auto‐segmentation model and a medical resident — both learned to delineate the CTV and OARs of cervical cancer from the same senior physician. This study demonstrates that in terms of both accuracy and efficiency, the deep learning‐based auto‐segmentation model was as accurate as the medical resident but with a much better computational efficiency. Furthermore, the auto‐segmentation approach offers additional perceivable advantages of being consistent and ever improving when compared with manual approaches. When carefully validated and implemented clinically, such as deep learning‐based method has the potential to improve the RT workflow.

## AUTHOR CONTRIBUTION STATEMENT

Zhi Wang, Xi Pei, and X. George Xu contributed to conception and design. Yin Lv, Weijiong Shi, and Fan Wang contributed to the source of datasets. Zhi Wang, Yankui Chang, and Zhao Peng contributed to auto‐segmentation model. Zhi Wang, Yankui Chang, Zhao Peng,, Xi Pei, and X. George Xu contributed to writing of the paper.

## CONFLICT OF INTEREST

No conflict of interest.
